# Development and characterization of antioxidant bilayer film based on poly lactic acid-bitter vetch *(Vicia ervilia)* seed protein incorporated with *Pistacia terebinthus* extract for active food packaging

**DOI:** 10.1016/j.crfs.2023.100613

**Published:** 2023-10-05

**Authors:** Sona Dodange, Hajar Shekarchizadeh, Mahdi Kadivar

**Affiliations:** Department of Food Science and Technology, College of Agriculture, Isfahan University of Technology, Isfahan, 84156-83111, Iran

**Keywords:** PLA, Bitter vetch seed protein, *Pistacia terebinthus* extract, WVP, Antioxidant capacity, Active food packaging

## Abstract

This study focuses on designing an active bilayer food package film based on polylactic acid (PLA) and bitter vetch seed protein incorporated with *Pistacia terebinthus* extract (PTE). The effect of PTE on the physicochemical, barrier, structural, mechanical, and antioxidant properties of the active film was determined. Moisture content, water solubility, and water vapor permeability (WVP) of the active films indicated that the addition of PTE increased its suitability for food packaging. FE-SEM micrographs illustrated that the resulting films had a smooth and dense surface, describing a continuous network of protein molecules within the film structure. FTIR analysis displayed the physical interaction between PTE and the film polymer. XRD revealed an increase in the crystallinity of the active films. The resulting active film had a low migration rate (<7%) of phenolic compounds into fatty food simulant. Notably, the addition of PTE significantly (P ≤ 0.05) decreased the tensile strength and Young's modulus (from 15.13 and 315.98 MPa to 14.07 and 254.07 MPa, respectively). Concurrently, there was an increase in the elongation at break of the active films (from 23.19 to 75.60%), indicating higher flexibility compared to control films. Additionally, the incorporation of PTE improved the thermal properties of active films. The antioxidant capacity of the designed films was measured based on their DPPH radical scavenging activity, revealing that the antioxidant capacity of the control film increased from 44.65% to 59.72% in the active film containing 15% PTE. In conclusion, the prepared bilayer film can effectively be used as an active food package for sensitive foods to oxidation.

## Highlights

• A bilayer film was designed based on poly lactic acid-bitter vetch seed protein.
• *Pistacia terebinthus* extract was added as a natural antioxidant to the bilayer film.
• Prepared active film showed excellent antioxidant activity.
• Active bilayer films showed better barrier and mechanical properties.
• Prepared active bilayer films can be used for packaging sensitive foods to oxidation.

## Graphical abstract

**Figure:**
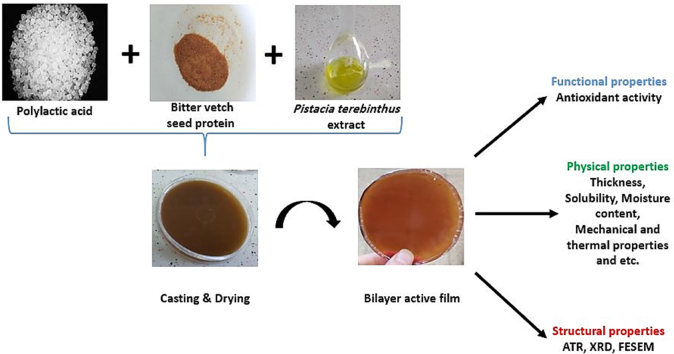

## 1 Introduction

Nowadays, active packaging is used for different foodstuffs to prevent oxidative, enzymatic, and microbial spoilage during shelf life (Azeredo et al., 2022). Bioactive and natural antioxidant materials are useful, inexpensive, and non-toxic materials to produce different active packaging systems (Moreira Gonçalves, Gomes Motta, Ribeiro-Santos, Hidalgo Chávez and Ramos de Melo, 2020). Proteins, polysaccharides, and lipids are the main solid matrix components for producing active edible packaging (Jafarzadeh et al., 2022). These components can operate as the carriers of bioactive compounds like oxygen scavenging, antimicrobial and antioxidant materials. Among these three groups, proteins are convenient because of their better blockage of gasses and mechanical attributes than the other two groups (Akrami et al., 2022).

Bitter vetch (*Vicia ervilia*) is a plant from the leguminous family and is usually cultivated in the Mediterranean climate. Despite having unique characteristics, including a high yield of proteins extraction, inexpensive source for proteins, ability to fix nitrogen, and finally, low nutritional requirements, this plant is usually used as a source of animal foods (Vioque et al., 2020). Seeds of bitter vetch (BV) contain nearly 25% protein. The protein of BV is a good source of essential amino acids like lysine, and its amino acid content is very similar to the soybean meal. Also, the protein of BV seeds is suitable for producing biodegradation films (Arabestani et al., 2016).

Polylactic acid (PLA), which is a linear aliphatic polyester, is produced by the fermentation of plant resources. Starch is changed to dextrose during PLA production. Then, lactic acid is generated from dextrose fermentation by the polycondensation process (Wang et al., 2023). The second method for PLA production, which is more common and leads to a higher-molecular-weight polymer, is lactide polymerization with the ring-opening process (Bonwick et al., 2019). Produced films from PLA have an excellent barrier and mechanical properties compared to synthetic polymers. However, it is recommended to use PLA in combination with other polymers because of its brittleness (Ding et al., 2022).

Natural polymers usually have weak mechanical properties and high permeability of gases. Some strategies have been suggested to solve these problems, including lamination. Lamination can improve the weakness of natural polymers by producing sheets from several layers on the natural polymer (Wu et al., 2021). For example, bilayer films from PLA and tilapia fish gelatin (FG) improved water vapor permeability and oxygen barrier properties compared to PLA and FG monolayers (Chen et al., 2022). Despite some favorable characteristics of bitter vetch seed protein, it also presents challenges, including weak mechanical strength and poor water vapor permeability (Arabestani et al., 2016). To improve these properties of the protein film, we explored the potential of bilayer films. In this research, we chose PLA due to its excellent barrier properties against water and superior tensile strength, aiming to counteract the limitations of protein films.

*Pistacia terebinthus*, also called the terebinth, is a member of the Anacardiaceae family and grows in the Mediterranean climate (Rauf et al., 2017). All parts of terebinth trees have been applied as useful therapies for different illnesses. The extract of terebinth fruits contains different bioactive materials, including essential fatty acids, antioxidants, and tannin. These unique properties of P. terebinthus extract make it a good source for producing active packaging films (Kaya et al., 2018).

This study aimed to prepare active bilayer films based on PLA/bitter vetch seed protein (BVSP) incorporated with ethanolic extracts of *Pistacia terebinthus* as a natural antioxidant compound and to investigate the structural, biological, and physicochemical properties of the prepared bilayer films.

## 2 Materials and methods

### 2.1 Materials

Bitter vetch seeds and *Pistacia terebinthus* were bought from local markets. Pellets of polylactic acid (4032D, extrusion grade, Mn = 88,500 g/mol and Mw/Mn = 1.8) were purchased from Nature Work Co. Ltd. (Blair, NE, USA). Glycerol, chloroform, gallic acid, sodium carbonate, and Folin-ciocalteau reagent were obtained from Merck Co. (Darmstadt, Germany). 2- phenol-1,1-hydrazine (DPPH) was bought from Sigma Chemical Co. (St. Louis, MO). All chemical materials had an analytical grade.

### 2.2 Extraction of *Pistacia terebinthus* extract (PTE)

Extraction was carried out according to Li et al. (2006) method with little modifications. *Pistacia terebinthus* was dried, powdered, and mixed with ethanol (70%) at the 1:7 ratio. Extraction was performed in a dark place for 48 h while stirring. Then, centrifugation at 3000 rpm for 20 min and filtration with Whatman filter paper (No. 41) was done. A rotary evaporator was used at 45 °C to concentrate the extract. Finally, the PTE was vacuum oven dried at 30 °C for 48 h and kept in the dark container at −18 °C.

### 2.3 Preparation of bilayer film containing PTE

Method of Kamali et al. (2022) with some modifications was used for the preparation of bilayer films. Firstly, 2 g PLA pellets was mixed with 100 mL chloroform for 8 h while stirring. Then, the mixture was casted on the glass petri dishes. The drying process of the first layer was lasted for 4 h at ambient temperature. Then, the second layer was formed on the dried PLA layer. For the preparing of second layer, bitter vetch seed protein (BVSP) solution (3% w/v) was prepared with distilled water, and the mixture was stirred for 1 h after pH adjustment to 11. Stirring was continuted at 85 °C for another 20 min for the protein denaturation. Then, glycerol (40% based on BVSP weight) as plasticizer and PTE at various concentrations (0, 5, 10 and 15% based on BVSP weight) were added and stirred for 1 h. Finally, the resulting dispersion was casted on PLA layer and dried at room temperature (25 ± 3 °C) and room relative humidity (35 ± 3%) for 3 days. The prepared films were conditioned at a desiccator by 55% relative humidity and room temperature for 48 h.

### 2.4 Characterization of bilayer films containing PTE

#### 2.4.1 Thickness, moisture content, water solubility, and water vapor permeability

The films thickness was calculated with a micrometer (0.001 mm sensitivity) (Mitutoyo-Co, Japan). The thickness of films was measured in ten various points of each film randomly (da Nóbrega Santos et al., 2022).

To determine moisture content of films, the weight of 2 × 2 cm^2^ samples of the films before (W_i_) and after (W_d_) oven drying at 105 °C for 24 h was measured. Moisture content was determined using Eq. (1) (Liu et al., 2021):

(1) $$\text{Moisture}\;\text{content}\;\left(\%\right)=\left[\left(W_{i}-W_{d}\right)/W_{i}\right]$$

To measure water solubility of films, the weight of 2 × 2 cm^2^ samples of the films was determined after oven drying at 105 °C for 24 h (Wi). Then, the film samples were soaked in 30 mL distilled water and shaked with shaker incubator (IKA KS 4000 I control, Germany) for 18 h at room temperature with 200 rpm. Filtration was done for separating insoluble samples by Whatman filter paper N. 41. Then insoluble samples were oven dried at 105 °C and weighted (W_f_). The solubility of the films was determined by Eq. (2) (Rangaraj et al., 2021):

(2) $$\text{Water}\;\text{solubility}\;\left(\%\right)=\left[\left(W_{i}-W_{f}\right)/\;W_{i}\right]\times 100$$

Permeability of films to water vapor was calculated according to the ASTM E96-95 standard method (ASTM, 1995). At first 3 g CaCl_2_ (RH = 0%) was poured in cups (6 cm × 1.5 cm) and film samples were attached to cups lid having a hole with a diameter of 8 mm. The cups were weighed and positioned into a desiccator with 100% relative humidity at room temperature. The weight of cups measured every day during a week. The water vapor permeability was measured by Eq. (3):

(3) WVP=WVTR×X/AΔP

where, WVTR shows transmission rate of water vapor (g/s), x is films mean thickness (m), A is area of lids hole (m^2^) and ΔP is water vapor pressure difference between desiccator and anhydrated CaCl_2_ in cups (Pa).

#### 2.4.2 Color and opacity

A colorimeter (Nippon Denshoku ZE 6000, Japan) was used to determine the color parameters (darkness/lightness (L*), redness/greenness (a*), and yellowness/blueness (b*)) of the films (Gürler et al., 2020). Color changes of designed active films was determined by calculating ΔE using Eq. (4) (Shi et al., 2023):

(4) $$\Delta E=\sqrt{{\left(L_{0}^{*}-L^{*}\right)}^{2}+{{\left(a_{0}^{*}-a^{*}\right)}^{2}+\left(b_{0}^{*}-b^{*}\right)}^{2}}$$

where L_0_*, a_0_*, b_0_*, L*, a*, and b* show the color parameters of control and active films, respectively.

Spectrophotometer (Nippon Denshoku, Japan) at 600 nm was used for 1 cm × 4 cm film samples to determine the opacity of the films using following Eq. (5) (Sogut, 2020):

(5) $$\text{Opacity}=A_{600\;\text{nm}}/t$$

where A_600nm_ is absorbance at 600 nm and t is the film thickness (mm).

#### 2.4.3 Mechanical properties

Tensile strength (TS), elongation at break (EB), and Young's modulus (YM) of the films were determined by a Universal Testing Machine (Bongshin, DBBP-20, Korea). 4.5 cm × 1.5 cm samples of the films were located among machine grips and mechanical properties were determined at 45 mm initial separation, 50 mm/min speed, and 20 kN tensile load at ambient temperature using the following Eqs. (Arrieta et al., 2022):

(6) $$\text{Tensile}\;\text{strength}=F_{\max}/A$$

(7) $$\text{Elongation}\;\text{at}\;\text{break}=\left(L_{\max}/\;L\right)\times 100$$

(8) $${\text{Young}}^{\prime }s\;\text{modulus}=\left(F.L_{0}\right)/\;\left(A.\;\Delta L\right)$$

where F_max_ shows peak load (N), A expresses area (m^2^), L_max_ is maximum length at rupture point (m), L_0_ shows initial length of prepared samples (m), F displays force (N) and ΔL is length changes (m).

#### 2.4.4 Antioxidant activity, polyphenol content, and migration of PTE

Method of Wu et al. (2019) was used to measure antioxidant activity of films with little modifications according to DPPH radical scavenging assay. For this purpose, 20 mg of each film samples were soaked in 4 mL ethanolic solution of DPPH (100 μM) and located in dark place for 30 min. Then, absorbance of each sample solution was determined with spectrophotometer at 517 nm. Finally, the antioxidant activity of films was determined by Eq. (9).

(9) $$\text{DPPH}\;\text{scavenging}\;\left(\%\right)=\frac{\left(A_{DPPH}-A_{s}\right)}{A_{DPPH}}\times 100$$

where A_DPPH_ and A_S_ show the absorption of DPPH and sample solutions, respectively.

80 mg of each film was mixed with 5 mL distilled water and shaked at 25 °C for 24 h. Then, supernatant was used to determine polyphenol content. Folin-Ciocalteu method was used to measure total polyphenols content (Agarwal et al., 2021). 2 mL Folin-Ciocalteu reagent (1:10 diluted) was mixed with 400 μl of sample. Then, 1.6 mL sodium carbonate 7% (w/v) was added, and after 30 min, the absorbance was determined at 760 nm. Gallic acid was used as the standard.

Water and 95% ethanol are introduced as aqueous and fatty food simulants, according to the European Commission (EC). The method of Busolo and Lagaron (2015) was used to determine migration of PTE. Ethanol (95%) and water were used as model foods. 2 × 2 cm^2^ samples of the films were soaked in 5 mL of water and ethanol and shaked at 25 °C for 7 days. Then, the migration of PTE was measured by measuring total polyphenols content.

#### 2.4.5 Morphology

Surface and cross-section images of films were prepared using a field emission-scanning electron microscopy (FE-SEM) microscope (Zeiss, Sigma, Germany). Films were broken in liquid nitrogen for taking cross-section photos. The samples were located in an aluminum holder, and the sputtering of films was performed with a gold layer.

#### 2.4.6 ATR-FTIR analysis

The presence of specific functional groups of films was investigated by attenuated total reflectance-Fourier transform infrared (ATR-FTIR) spectroscopy using an ATR-FTIR spectrophotometer (Bruker model Tensor 27, Germany) at a wavenumber range of 600–4000 cm^−1^.

#### 2.4.7 Crystalline structure

An X-ray diffractometer (AXS Analytical X-Ray, Siemens D 5005, Germany) was used to recorde X-ray diffractogram (XRD) patterns of films. After locating samples inside a frame, the reflected beam was measured by radiation from a copper anticathode with CuKα = 1.54178 Å wavelength at 40 mA current and voltage of 40 kV in the range of 2θ = 10–100°.

#### 2.4.8 Thermal analysis

Differential scanning calorimetry (DSC) was performed with an STA device (Netzsch STA 449F3 Jupiter®, Germany). The film sample (5 mg) was placed in an aluminum pan, and the heating process was carried out from 25 °C to 500 °C with a heating rate of 10 °C/min. Thermogravimetric analysis (TGA) was also investigated by STA device at 25–500 °C and a heating rate of 10 °C/min in a nitrogen atmosphere.

### 2.5 Statistic analysis

The effects of *Pistacia terebinthus* extract addition on the properties of the designed bilayer films based on PLA/BVSP were investigated in a completely randomized design. All experiments were carried out in three replicates. ANOVA method and SAS software were used for statistical analysis. Duncan's multiple tests (p < 0.05) used to compare the mean values.

## 3 Results and discussion

### 3.1 Thickness, moisture content, water solubility, and WVP

Thickness is an important film property because it affects other properties, like permeability and mechanical properties (Shahrampour et al., 2020). Results of active films thickness are shown in Table 1. Results showed that addition of 5 and 10% of PTE to protein layer of bilayer films did not have significant effect on thickness. But films with 15% PTE were significantly (P ≤ 0.05) thicker than other three films due to higher solid components entering to the film matrix (Tongnuanchan et al., 2014).

**Table 1:** Thickness, solubility, moisture, and WVP of films.

Sample	Thickness (mm)	Moisture content (%)	Solubility (%)	WVP (g.m/Pa.s.m^2^)
0% extract	0.32 ± 0.02^b^	12.23 ± 0.41^a^	24.95 ± 1.69^a^	5.47E^−10^ ± 1.18E^−10a^
5% extract	0.33 ± 0.01^b^	10.53 ± 0.6^b^	24.32 ± 0.82^a^	5.25E^−10^ ± 1.05E^−10a^
10% extract	0.34 ± 0.01^b^	8.91 ± 0.33^c^	23.45 ± 0.28^b^	3.44E^−10^± 1.07E^−10b^
15% extract	0.36 ± 0.01^a^	8.90 ± 1.18^c^	21.29 ± 0.21^c^	3.19E^−10^ ± 9.15E^−10b^

Different small letters indicate significant differences between samples (P < 0.05).

Results of the moisture content, water solubility, and WVP of active films are also shown in Table 1. These results express that adding PTE to the bilayer films significantly decreased moisture content, water solubility, and WVP of active films. It should be due to the formation of new hydrogen bonds between protein and PTE that can limit the formation of hydrogen bonds between water molecules and protein. Forming hydrogen bonds between the extract and protein reduces available amino and hydroxyl groups of BVSP, so hydrophile groups decrease (Hafsa et al., 2016). These results show that the addition of PTE made the prepared active films more hydrophobic and more suitable for food packaging.

### 3.2 Color and opacity

The film color and opacity are important in the food packaging industry owing to their effects on the consumer's acceptance (Jeevahan et al., 2020). The color and opacity of the active films are shown in Table 2. Results show that the incorporation of PTE in the films significantly (P ≤ 0.05) changed color and opacity. The addition of PTE to films decreased L* and increased the opacity of active films, probably due to the refraction of light with droplets of extracts. Darker films are good choice for sensitive foods to light (Zhang et al., 2022). Table 2 also indicates a significant (P ≤ 0.05) increase of a* and b* values after incorporating PTE in the film. Overall, color changes of active films increased from 3.69 to 10.55 by increasing PTE concentration from 5 to 15% in active films matrix. Natural pigments in extracts are responsible for the color changes of active films. Some of these pigments are carotenoids, betacyanins, betalamic acid and flavonoids (Kanatt, 2020). Similar results were reported by Jamróz et al. (2019) that showed a reduction of film lightness but an increase of a* and b* values after adding tea polyphenols to chitosan films comparing to control films. Also, Kaya et al. (2018) reported that darker color and more opacity was observed in active films containing extract of *Pistacia terebinthus* in comparison to chitosan film because of pigments of *Pistacia terebinthus* extract.

**Table 2:** Color parameters and opacity of films.

Sample	a*	b*	L*	ΔE	Opacity (UA/μm)
0% extract	27.75 ± 1.22^d^	53.13 ± 0.75^c^	39.58 ± 2.35^a^	–	2.55 ± 0.08^d^
5% extract	29.43 ± 0.47^c^	56.39 ± 0.24^b^	39.18 ± 0.52^a^	3.69 ± 0.18^c^	2.61 ± 0.06^c^
10% extract	31.58 ± 2.12^b^	56.89 ± 0.71^b^	37.66 ± 1.10^b^	5.70 ± 0.35^b^	2.84 ± 0.02^b^
15% extract	36.81 ± 0.57^a^	57.99 ± 0.85^a^	37.23 ± 0.08^b^	10.55 ± 0.59^a^	2.95 ± 0.02^a^

Different small letters indicate significant differences between samples (P < 0.05).

### 3.3 Mechanical properties

The flexibility and elasticity of packaging films are important factors in food packaging. Mechanical properties of the films, including TS, EB, and YM, were determined to investigate the mechanical properties of active films, which are expressed in Table 3. The amounts of TS, EB, and YM of control bilayer films show that the control bilayer film was firm, rigid, and brittle. But the addition of PTE to the film significantly (P ≤ 0.05) decreased TS and YM of active films, while EB of active films increased, showing higher flexibility of the active films compared to control films. The presence of PTE in active films causes changes in the film structure that cause changes in the mechanical properties of the active films. PTE prevents the formation of a strong network matrix and interaction between BVSP polymers through their interaction with proteins. It has been reported that additive compounds, except cross-linking agents, decrease film TS (Cagri et al., 2001). Similar results were reported by Wang et al. (2021), which showed a reduction of films TS after the addition of bamboo leaf volatile oil to corn starch-based films. Also, Nunes et al. (2021) reported similar behaviors for the films based on gelatin incorporated with lemon essential oil. They expressed a reduction in film resistance to tension because of forming a complex between the polymer molecules and essential oils, which weakens the polymeric network.

**Table 3:** Mechanical properties of films.

Sample	TS (MPa)	EB (%)	YM (MPa)
0% extract	15.13 ± 0.88^a^	23.19 ± 3.87^c^	315.98 ± 5.28^a^
5% extract	15.09 ± 0.60^ab^	51.54 ± 4.11^b^	300.93 ± 1.19^a^
10% extract	14.63 ± 0.13^b^	60.63 ± 6.50^b^	274.05 ± 12.75^b^
15% extract	14.07 ± 0.13^c^	75.60 ± 12.53^a^	254.07 ± 13.70^c^

Different small letters indicate significant differences between samples (P < 0.05).

### 3.4 Antioxidant activity, polyphenols content, and migration of PTE

Active packages with antioxidant activity improve the shelf life of the foods by controlling oxygen levels of food packages and decreasing the rate of food spoilage reactions (Vuong et al., 2013). The results of polyphenol contents and antioxidant activity of the bilayer film are presented in Table 4. As expected, higher amounts of PTE caused higher polyphenol contents and antioxidant activities of active films. Results also showed that the control film had antioxidant activity because BVSP contains high levels of aromatic amino acids, which can react with free radicals through their side chains. Also, basic and acidic amino acids of BVSP, which can chelate ions of metals, cause antioxidant activity in control films (Rajapakse et al., 2005). The enhanced antioxidant activity observed in active films containing PTE can likely be attributed to its rich phenolic compound content and notable hydrogen-donating capacity, as reported by Kaya et al. (2018). Additionally, Djebari et al. (2021) highlighted a significant presence of phenolic compounds in multilayer active films based on LDPE/PET that incorporate the extract of *Pistacia lentiscus* L.

**Table 4:** Polyphenol content, antioxidant activity, and migration of films.

Sample	Polyphenol content (mg GAE/g dried film)	DPPH radical scavenging activity (%)	Migration test	
			Food model	Polyphenol content (mg GAE/kg simulant)
0% extract	8.02 ± 1.66^d^	44.65 ± 0.76^c^	Water	–
			Ethanol 95%	–
5% extract	20.35 ± 1.77^c^	48.30 ± 1.90^c^	Water	15.76 ± 2.75^c^
			Ethanol 95%	6.61 ± 0.11^b^
10% extract	57.89 ± 7.10^b^	54.41 ± 3.26^b^	Water	42.32 ± 0.13^b^
			Ethanol 95%	6.85 ± 0.03^a^
15% extract	96.80 ± 1.12^a^	59.72 ± 0.94^a^	Water	74.00 ± 0.14^a^
			Ethanol 95%	6.92 ± 0.06^a^

Different small letters indicate significant differences between samples (P < 0.05).

Active packages play their roles by releasing active compounds on the surface of foods. Therefore, the migration test is an important test to determine the releasing amounts of active compounds. FDA (2007) introduced water and 95% ethanol as aqueous and fatty food simulants, respectively, to study the migration of active compounds. Releasing of phenolic compounds from active films into the water was significantly higher than 95% ethanol owing to solubility in the water of the active film. Tran et al. (2017) expressed that various factors could affect releasing active compounds into foods, including diffusion of liquids in the film structure, film solubility in the food simulants, and film diffusion into food simulants. On the other hand, the polarity of the active compounds and food simulants also affects the migration of active components.

### 3.5 Morphology

**Fig. 1:**
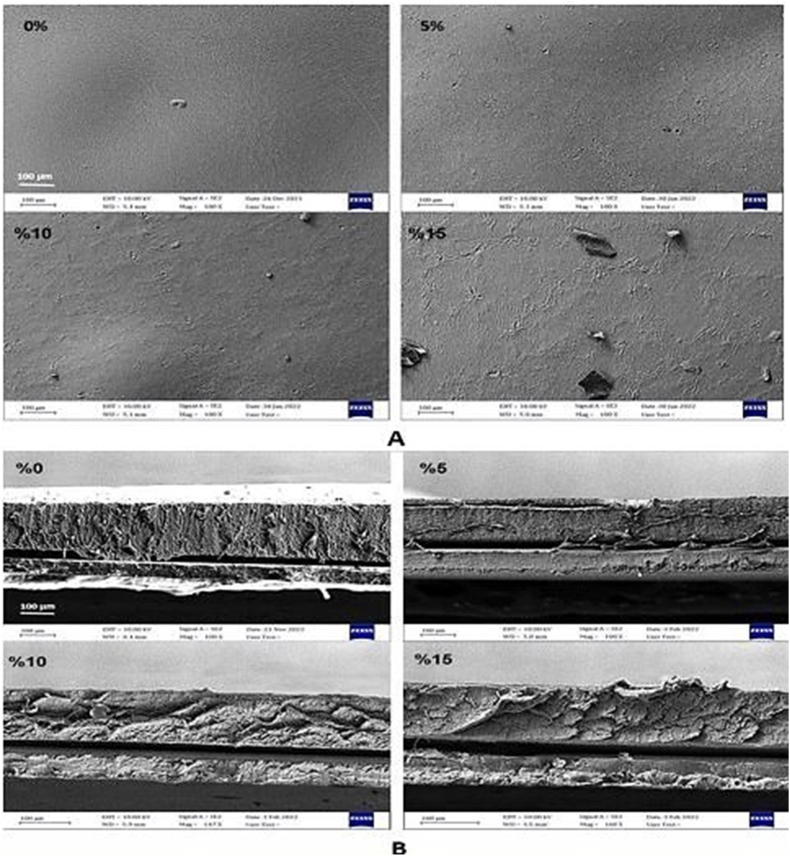
FE-SEM images of surface (A) and cross-section (B) of films (control and active bilayer films).

Homogeneity and morphology of the film structure can affect the permeability of the film samples. The surface and cross-section FE-SEM micrographs of the control and active films (incorporated with PTE) are illustrated in Fig. 1. A and B, respectively. The inactive bilayer films showed a smooth and dense surface, indicating the formation of a continuous network of protein molecules in the film structure. This compact surface was also observed in active films containing 5 and 10% PTE, although some grains can be seen after incorporating PTE into the films. The incorporation of the highest amount of PTE caused larger grains in the film. A low concentration of the extract was dispersed well in the film matrix, which caused a uniform film structure. More grains were observed and caused a more nonhomogeneous surface by increasing the PTE concentration. Similar results were reported by Riaz et al. (2020). The cross-section micrographs, in which the protein layer is at the top and PLA at the bottom, showed a compact structure of films. However, some differences were observed in active films depending on the amount of PTE. The active films with lower incorporated PTE had a similar matrix and structure to the control film sample. However, the compactness of the film decreased in the active films. These changes could be due to the disruption of the film network by polyphenols molecules during drying. Similar results are reported for other active films like carrageenan/SEO (Shojaee-Aliabadi et al., 2013).

### 3.6 ATR-FTIR analysis

**Fig. 2:**
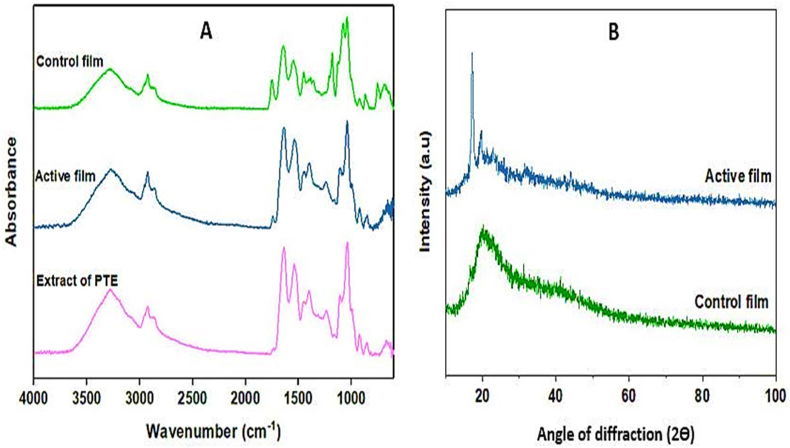
ATR-FTIR spectra (A) and XRD patterns (B) of PTE, active and control films.

ATR-FTIR spectra of PTE, control film, and active films are shown in Fig. 2A. The spectrum of active film shows a broad band at 3280 cm^−1^, which is related to O–H stretching vibration (Martins et al., 2018). The bands at 2873 and 2925 cm^−1^ are assigned to C–H stretching vibrations of CH_3_ and CH_2_ groups, respectively (Nilsuwan, Guerrero, de la Caba, Benjakul and Prodpran, 2020). The bands at 1635 and 1541 cm^−1^ are related to C=O stretching vibration coupled with the C–N and N–H bending, respectively (Scaffaro et al., 2018). The band at 1724 cm^−1^ might be related to stretching vibration of NH_2_ and C=O. Another band at 1758 cm^−1^ is due to the typical asymmetric stretching vibration of carbonyl group (C=O) in PLA film (Martins et al., 2018). Other bands at 1452 and 1386 cm^−1^ are assigned to C–C stretching. There are absorbance bands at about 1037 and 1232 cm^−1^ that are related to the OH group of glycerol (Ali et al., 2019). The band at around 811 cm^−1^ is related to aromatic C–H vibration (Ardjoum et al., 2021).

The FTIR spectrum of PTE shows an absorbance band at 3300 cm^−1^ related to O–H stretching vibration. The presence of this band is related to phenolic groups of PTE (Martins et al., 2018). There are bands at 2800 and 2900 cm^−1^, which are related to C–H and N–H stretching vibration, respectively (Valdés et al., 2021). The sharp bands at around 1600 - 1750 cm^−1^ are due to stretching vibration of C=C and C=O bonds and asymmetric bending of N–H, which are characteristic to amino acids and flavonoids (Ardjoum et al., 2021). Another band at 1263 cm^−1^ is related to C–O group of polyols, like hydroxyflavonoids (Valdés et al., 2021). Other band at 1323 cm^−1^ is assigned to C–C vibration of aromatic rings. The sharp bands at 1088 and 1200 cm^−1^ are characteristic to the C–O bending of ester group. As can be seen, no new peak was created after the addition of PTE to control bilayer film. However, some bands are shifting in the FTIR spectrum of active films. These results show the physical, not chemical, interaction of PTE with film polymer.

### 3.7 Crystalline structure

The XRD patterns of the films are illustrated in Fig. 2B. The XRD pattern of control bilayer film showed a peak at 2θ = 20°, indicating the amorphous structure of the control film. Peak intensity at 2θ = 20° decreased after addition of PTE to control film. Also, two sharp peaks were observed at 2θ = 18 and 21° due to development of new hydrogen bonds and crystallinity increase of the active films. Zhou et al. (2021) also reported these results for cassava starch film containing cinnamon essential oil.

### 3.8 Thermal analysis

**Fig. 3:**
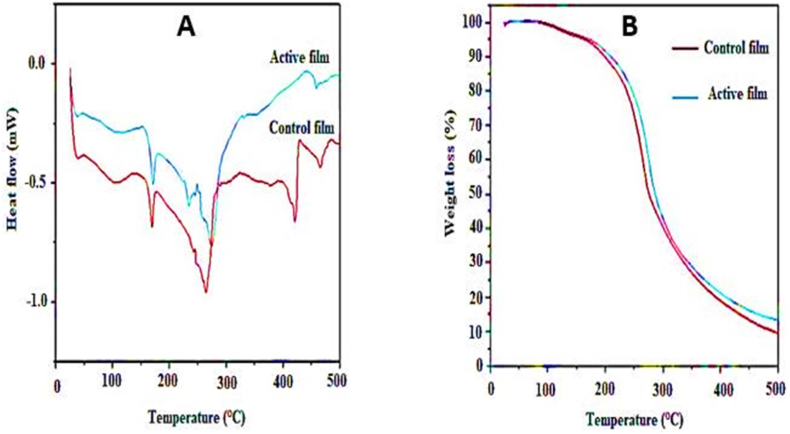
DSC (A) and TGA (B) curves of control and active bilayer films.

Thermal analysis was done to evaluate the effect of extract addition on the thermal stability of the bilayer film. The melting and decomposition temperatures of bilayer films containing PTE are shown in Fig. 3A. The glass transition of the control film was 100 °C. The control bilayer film showed a melting temperature of 190 °C and a decomposition temperature of 440 °C. Addition of extract to the control film did not exhibit a significant effect on the DSC curve of the bilayer film; however, the melting temperature of the active film shifted to 200 °C. Also, the decomposition temperature of the active film increased to 460 °C, confirming the increase of thermal stability of active film due to the hydrophobic nature and high molecular weight of PTE. Similar results were reported by Roy and Rhim (2020) for PLA film containing curcumin essential oil.

TGA curves of control and active films are shown in Fig. 3B. Three main weight loss steps were observed for the both films. The first step of weight loss occurred at about 80–170 °C, which was related to evaporation of water and residual solvent in the structure of films. The second step occurred at 170–260 °C due to the disintegration of glycerol. The third step, which occurred in 260–420 °C, was related to the decomposition of polymers. So, the highest amount of weight loss happened in this step. TGA curves showed that active film had more thermal stability and lower weight loss than inactive film. Similar findings have been reported by Kanmani and Rhim (2014).

## 4 Conclusion

PTE was selected as a natural antioxidant agent for the preparation of active bilayer films in this study owing to its strong antioxidant activity. The properties of active bilayer films containing different amounts of extract (5, 10, and 15%) were compared with those of the control film. Analyses using ATR-FTIR, FE-SEM, and XRD confirmed that PTE was successfully integrated into the film structure. Notably, the active films demonstrated enhanced physical properties, such as thickness, water solubility, and moisture content, in comparison to the control film. Furthermore, they exhibited superior barrier characteristics, evidenced by their reduced water vapor permeability and heightened antioxidant activities. While the inclusion of PTE reduced the tensile strength of the active films, it led to a noticeable increase in their elongation at break. These results showed that the prepared active films containing 15% PTE with almost good mechanical properties, excellent resistance against water (low WVP and solubility in water), and high amounts of polyphenols and antioxidant activity are suitable for packaging foods that are sensitive to oxidation. More investigations are needed to standardize PTE for guarantee its safety and effectiveness.

## CRediT authorship contribution statement

**Sona Dodange:** Conceptualization, Experiment, Software, Writing – original draft. **Hajar Shekarchizadeh:** Conceptualization, Supervision, Visualization, Writing – review & editing. **Mahdi Kadivar:** Supervision, Visualization, Writing – review & editing.

## Declaration of competing interest

I testify on behalf of all co-authors that there are no known competing financial interests or personal relationships that could have appeared to influence the work reported in this paper.

## Data Availability

Data will be made available on request.
